# Procedures for measuring and verifying gastric tube placement in newborns: an integrative review

**DOI:** 10.1590/1518-8345.1841.2908

**Published:** 2017-07-10

**Authors:** Flávia de Souza Barbosa Dias, Suellen Cristina Dias Emidio, Maria Helena Baena de Moraes Lopes, Antonieta Keiko Kakuda Shimo, Ana Raquel Medeiros Beck, Elenice Valentim Carmona

**Affiliations:** 1 Doctoral student, Faculdade de Enfermagem, Universidade Estadual de Campinas, Campinas, SP, Brazil.; 2 Doctoral student, Faculdade de Enfermagem, Universidade Estadual de Campinas, Campinas, SP, Brazil. Scholarship holder at Coordenação de Aperfeiçoamento de Pessoal de Nível Superior (CAPES), Brazil.; 3 PhD, Full Professor, Faculdade de Enfermagem, Universidade Estadual de Campinas, Campinas, SP, Brazil.; 4 PhD, Professor, Faculdade de Enfermagem, Universidade Estadual de Campinas, Campinas, SP, Brazil.

**Keywords:** Intubation, Gastrointestinal, Infant, Newborn, Nursing

## Abstract

**Objective:**

to investigate evidence in the literature on procedures for measuring gastric tube insertion in newborns and verifying its placement, using alternative procedures to radiological examination.

**Method:**

an integrative review of the literature carried out in the Cochrane, LILACS, CINAHL, EMBASE, MEDLINE and Scopus databases using the descriptors “Intubation, gastrointestinal” and “newborns” in original articles.

**Results:**

seventeen publications were included and categorized as “measuring method” or “technique for verifying placement”. Regarding measuring methods, the measurements of two morphological distances and the application of two formulas, one based on weight and another based on height, were found. Regarding the techniques for assessing placement, the following were found: electromagnetic tracing, diaphragm electrical activity, CO_2_ detection, indigo carmine solution, epigastrium auscultation, gastric secretion aspiration, color inspection, and evaluation of pH, enzymes and bilirubin.

**Conclusion:**

the measuring method using nose to earlobe to a point midway between the xiphoid process and the umbilicus measurement presents the best evidence. Equations based on weight and height need to be experimentally tested. The return of secretion into the tube aspiration, color assessment and secretion pH are reliable indicators to identify gastric tube placement, and are the currently indicated techniques.

## Introduction

Insertion of Gastric Tube (GT) in Newborns (NB) hospitalized in the Neonatal Intensive Care Unit (NICU) is one of the most commonly performed nursing procedures. It is indicated for gastric decompression, administration of medications, and mainly for feeding the gastric tube process, and despite being a standard procedure for nurses working in the NICU, it is not risk free and involves decisions that may compromise patient safety^1^.

Some of the important aspects to increase safety in using GT in newborns involve care in measuring the insertion length, assessing placement/positioning of the distal end of the tube, and in maintaining its correct positioning^1^. Serious respiratory complications may occur due to bronchopulmonary aspiration of gastric contents or inadequate tube placement reaching the respiratory tract. Intestinal absorption problems and alimentary intolerance related to GT positioning in the pylorus or duodenum can also occur. Moreover, difficulties encountered in the trajectory can cause puncture injuries to the esophagus or respiratory tract^2^. The occurrence of errors in GT placement is very frequent: studies show proportions of 47.5 to 59% inadequate placement between neonatal and pediatric patients^3^ ^-^ ^4^.

The nurse’s decision-making process during gastric tube procedure begins with the choice of an effective method that has a strong association with measuring the actual tube route from the nostril or oral cavity to the body of the stomach, passing through the entire length of the esophagus^1^.

After choosing the measuring method and performing the insertion, it is necessary to verify that the distal end of the tube has reached the body of the stomach, as well as whether all the distal orifices are within the gastric cavity in order to prevent fluid leakage into the esophagus or duodenum^1^. 

Radiological examination of the chest and abdomen is considered the gold standard verification technique, since it allows visualization of the GT route and the positioning of its distal end. Despite presenting the most reliable result, this technique is costly and is not commonly used in neonatal clinical practice for this reason, as the GT is often replaced, and repeated exposure to radiation can be dangerous^2^. Another limitation is the fact that this test is only effective at the moment it is performed, since tube displacement can happen immediately after^2^ ^,^ ^5^, thus requiring the use of other techniques to assess tube placement other than radiological examination. 

In this integrative review, we sought evidence that may assist nursing assistants in the decision-making process regarding gastric tubes in newborns in the NICU, given the importance of always choosing the best health practices aiming at patient safety. Thus, this study aimed to investigate evidence in the literature on procedures for measuring gastric tube insertion in newborns and verifying its placement, using alternative procedures to radiological examination.

## Method

This is an integrative review of the literature which seeks to synthesize results from previous studies on the proposed subject^6^. Integrative reviews have the potential to evidence comprehensive understanding of specific issues and to identify gaps in knowledge. This is a very useful method for nurses who are in clinical practice and wish to perform nursing assistance based on scientific evidence^7^ ^-^ ^9^. 

The steps followed in elaborating this review were: establishing the research question, conducting a literature search, evaluating data, analysing the included studies, interpreting the results and presenting the review^8^. 

The guiding question of this study was “What are the procedures for measuring gastric tubes in newborns and for assessing its placement, other than radiological examination?”

The search was performed in January 2017 in the following databases: Cochrane Library, Cumulative Index to Nursing and Allied Health Literature (CINAHL), Excerpta Medica dataBase (EMBASE), Literature of Latin American and the Caribbean on Health Sciences (LILACS), Medical Literature Analysis and Retrieval System Online (MEDLINE) and Scopus. No time frame for inclusion of articles was established. 

The terms used in the searches were extracted from the Health Sciences Descriptors (DeCS) and from the Medical Subject Headings (MeSH), and included:** **Intubation, Gastrointestinal and Newborns, as well as their respective versions in Portuguese and Spanish. Synonymous terms suggested by EMBASE at the time of the search were also searched. In order to delimit the search, publications with the terms *gastrostomy, pain, surgery* and *intubation intratracheal* were excluded for not addressing the subject of this review. Publications contained in the references of the selected studies whose titles addressed the research subject were also investigated.

**Figure 1:**
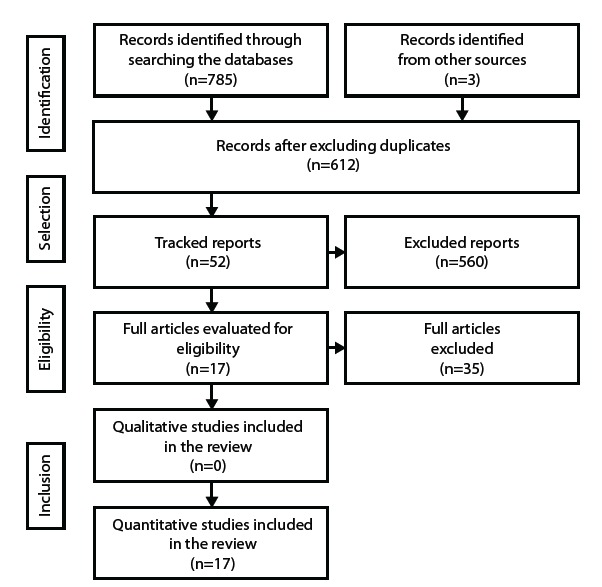
Flowchart of the identification, selection and inclusion process of the studies, elaborated based on the PRISMA recommendation^(10)^.

Article selection was carried out by two researchers independently, and inclusion criteria were: original studies published in-full that address, in the title or abstract, gastric tube measurement procedures and/or techniques for assessing its placement, and which included newborns in the studied sample; studies published in Portuguese, English or Spanish. Theses and dissertations, pilot studies, review articles, case or experience reports, letters, editorials and publications where the method was not clearly described were excluded. PRISMA recommendations^10^ were followed for the study selection, as shown in Figure 1. 

A form with the following items was elaborated by the authors for developing the analysis: bibliographic reference, level of evidence, language, country of origin, main researcher’s training, database, objective, study design, ethical considerations, subjects, main results, conclusion and limitations.

Seven (7) levels of classification were considered to categorize the level of evidence: level 1 - systematic review or meta-analysis of controlled clinical trials; level 2 - well-delineated randomized controlled clinical trial; level 3 - controlled clinical trial without randomization; level 4 - well-delineated cohort or case-control studies; level 5 - systematic review of qualitative and descriptive studies; level 6 - descriptive or qualitative studies; and level 7 - opinion of authorities or experts^11^. The results were analyzed and presented in a descriptive way.

As this is an integrative review, it was not necessary to request approval from the Ethics Committee to carry out the study. We declare no conflicts of interest.

## Results

**Figure 2:**
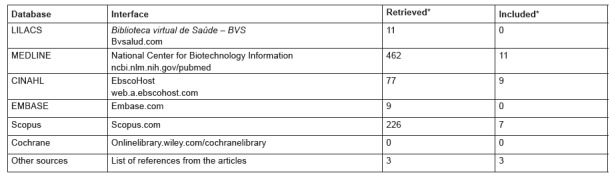
Number of publications found in the databases and included in the study.

The number of publications found in the investigated databases, as well as other sources included in this review are presented in Figure 2. 

* Some publications were repeated in more than one database

**Figure 3:**
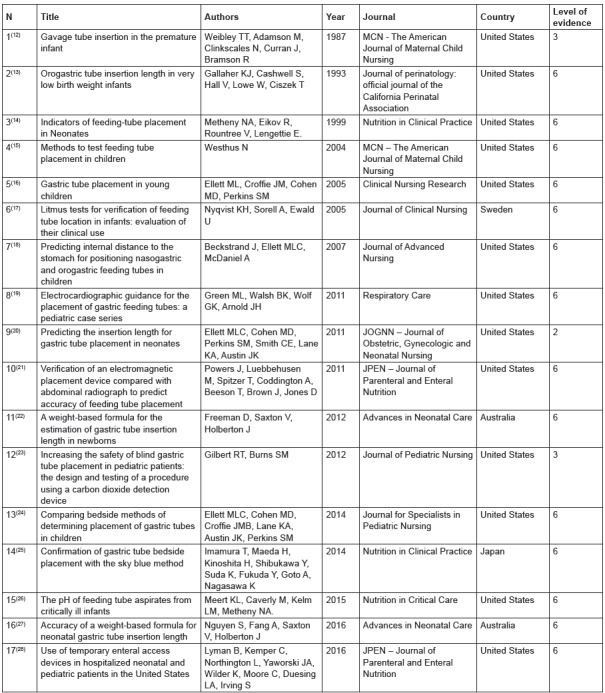
Characterization of publications and levels of evidence of the articles included in the review.

The 17 articles included in the review were all published in English between 1987 and 2016. The majority of the studies were carried out in the United States (n = 13), the main authors had training in nursing (n = 11) and medicine (n = 6). The included studies were classified into two categories for data analysis: “Methods for measuring gastric tube” and “techniques for assessing gastric tube placement”. Characterization of the articles considering the level of evidence is presented in Figure 3.

### Methods for measuring the gastric tube

**Figure 4:**
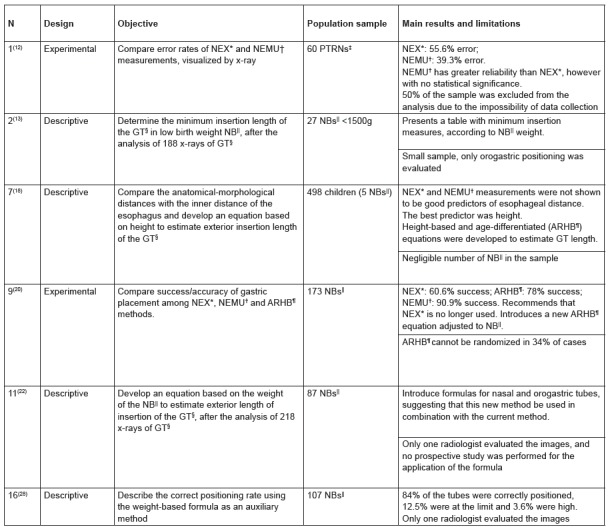
Studies on gastric tube measurement methods.

Among the articles that addressed GT measurement, four were observational studies^13^ ^,^ ^18^ ^,^ ^22^ ^,^ ^27^ and two were experimental studies^12^ ^,^ ^20^, and were mostly published in nursing journals. With regard to ethical aspects, only one article^12^ did not report having submitted the study to ethical appreciation. Figure 4 briefly describes each of these studies, addressing the design, objective, population sample, main results and limitations. 

*(*Nose, Earlobe, Xiphoid*): distance measured from the tip of the nose to the earlobe to the xiphoid process; †(*Nose, Earlobe, Mid-Umbilicus*): distance measured from the tip of the nose to the earlobe, a point halfway between the xiphoid process and the umbilicus; ‡pre-term newborns; §gastric tube; ||newborns; ¶(*Age Related, Height Based*): height-based equation classified by age.

The methods described in the literature for GT measurement in NBs include the NEX and NEMU morphological measures. NEX (Nose, Earlobe, Xiphoid) corresponds to the distance measured from the tip of the nose to the earlobe to the xiphoid appendix, while NEMU (Nose, Earlobe, Mid-Umbilicus) corresponds to the distance measured from the tip of the nose to the earlobe to a point halfway between the xiphoid process and the umbilicus^12^.

A method that determines the minimum insertion length of the tube has been specifically developed for low birth weight newborns (<1500g)^13^. Minimum insertion measures proposed in this study are 13cm for newborns weighing less than 750g, 15cm for newborns weighing between 750 and 999g, 16cm for newborns weighing between 1,000 and 1,249g, and 17cm for those weighing between 1,250 and 1,499g. Application of this minimum insertion length method to a sample of 27 NBs weighing less than 1,500 g showed an increase in the proportion of correct gastric tube positioning from 62 to 86%. This method makes it possible to avoid positioning the end of the tube above the gastreophageal junction, thus reducing the risk of aspiration and other respiratory complications.

In addition to these measurements, two equations are described to estimate the insertion length of the tube: the height-based equation^18^ ^,^ ^20^ and the weight-based formula^22^. According to one of the studies^18^ selected in this review, NEX and NEMU morphological measures do not present good predictors of the internal measurement due to their high variability when repeated measures are taken. 

In comparing several external measurements with internal measurement verified by endoscopy or esophageal manometry, the results showed that height was the best predictor for measuring the gastric tube. The relationship between height and internal measurement of tube passage varied according to age; therefore, specific equations at different age intervals were developed for calculating the insertion measurement of the naso-orogastric tube. When these equations were projected onto the studied sample through computational analysis, the performance was very promising, with success rates between 96.5 and 98.8%, depending on the infant’s age^18^. However, a major limitation of this study considering the objective of the present review was the small participation of NBs, with only 1% in the studied sample. 

A study comparing the accuracy/success rates of the NEX, NEMU methods and the height-based equation (ARHB - Age Related, Height Based) performed two different analyzes^20^. In the first analysis, the end of the tube visualized in the stomach, pylorus or duodenum was considered as correct positioning, and the accuracy ratio was 60.6% for NEX, 92.4% for NEMU and 100% for ARHB. NEMU and ARHB measurements were significantly higher than NEX (p<0.001). In the second, more restrictive analysis, only the tubes visualized in the stomach were considered to be positioned correctly. The results of the second analysis were: 60.6% accuracy for NEX, 90.9% for NEMU and 78% for ARHB. Although no significant difference (p = 0.615) between NEX and ARHB rates were found in the second analysis, it can be noticed that all errors presented by NEX measure occurred by placing the tube above the gastroesophageal junction, while the errors presented by the ARHB measure were always below the pylorus. This difference is relevant with respect to the type of error, its risks and complications. During this study, the authors also developed a new ARHB equation adjusted for use in newborns between 35 and 56.5cm in length for measuring the nasogastric tube: 1.95 +0.372x[height in cm]. It was not possible to develop a new equation for orogastric route in newborns with the mentioned length due to the small number tubes inserted by this route in the sample (10.4%)^20^. 

Another method described in the literature is the weight-based equation^22^. The authors justify the need to create this method based on the fact that height is not an easily accessible measure in neonatal clinical practice, while in contrast weight is a more viable predictor as it is checked daily and used as a reference for several clinical applications such as calculation of drug dosages, diets and estimating catheter insertion, among others. In this study, 218 radiological images were analyzed, and by way of using a linear regression analysis, formulas for orogastric (3x[weight in kg]+12) and nasogastric tubes (3x[weight in kg]+13) were developed. When designing such formulas in the studied sample based on computational analysis, it was possible to predict 100% of poorly placed nasogastric and 60% orogastric tubes. The lower rates found in orogastric tubes may be related to the fact that the tubes move more when positioned in the oral cavity. 

The use of the weight-based formula as an auxiliary method to NEMU in GT insertion was described in another study^27^, however, the result was lower than expected, with 16% of tubes being incorrectly positioned (above or near the gastroesophageal junction). The authors suggest that this result is justified by the fact that the formula was not fully incorporated by the nursing team as a measurement strategy. When individually analyzing the 31 cases of incorrect positioning, 22 (71%) of them would have been avoided if the formula had been calculated and used.

### Techniques for assessing gastric tube placement

**Figure 5:**
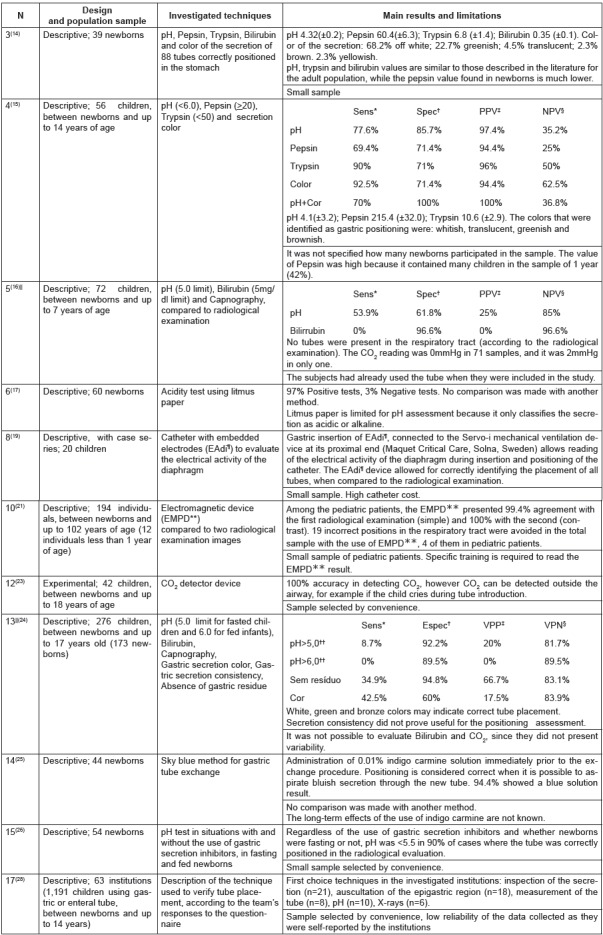
Studies on techniques for assessing gastric tube placement.

Of the 11 studies classified in this category, 10 were observational studies that investigated alternative techniques to visualizing radiological imaging, established as the gold standard to verify GT placement. Such alternative techniques have the objective of improving patient safety, achieving a reduction of radioactive exposure without increasing the risk and complications related to incorrect tube placement. The studies included in this category are described in detail in Figure 5.

*Sensitivity; †Specificity; ‡Positive predictive value; §Negative predictive value; ||Investigated the accuracy to determine incorrect positioning; ¶Electrical Activity of the Diaphragm; **Electromagnetic Placement Device; †† Only refers to NBs included in the sample.

The techniques investigated to verify GT positioning in NBs include: gastric secretion aspiration; epigastric region auscultation; checking aspirated secretion’s pH, pepsin, trypsin and bilirubin; secretion color; presence of CO_2_ test; acid test with litmus paper, reading diaphragm’s electrical activity; electromagnetic tracing and the use of indigo carmine at 0.01%. 

The diagnostic accuracy tests used in three studies^15^ ^,^ ^16^ ^,^ ^24^ included in this review were always compared to radiological examination. However, one study^15^ evaluated the test accuracy in identifying correctly positioned tubes, and two other studies^16^ ^,^ ^24^ evaluated the accuracy in identifying incorrectly positioned tubes. This prevents the simple comparison of the values between the three studies. 

The study that investigated the accuracy of correctly positioned tubes found that the use of pH evaluation along with color evaluation is the safest technique to confirm correct positioning, considering pH <6.0 and translucent greenish and brownish colors^15^. 

For studies that performed accuracy tests for incorrect positioning of the tube^16^ ^,^ ^24^, the most important value to be considered is positive predictive value, since the use of the investigated techniques occurs at the bedside and represents the proportion of tests that assertively indicate incorrect positioning of the tube. The indicator with the highest positive predictive value (66.7%) was absence of aspirated secretion. The second most important indicator was the pH test, which presented positive predictive values ranging from 20 to 25%.

The accuracy of capnography in identifying incorrect positioning of the GT cannot be confirmed as there were no placements in the respiratory tract^16^ ^,^ ^24^, and also because it is possible to detect the presence of CO_2_ in the oral cavity, oropharynx, esophagus and stomach^23^. 

The evaluation of bilirubin presence was not a reliable indicator to identify incorrect positioning, since it did not predict tubes positioned in the duodenal portion^(16, 24)^. 

The use of the electromagnetic tracing device and evaluating electrical activity in the diaphragm showed good precision and accuracy. The major advantage of these techniques is the possibility of real-time path correction during tube passage, as well as avoiding exposure to radiation, since these procedures are presented as possible substitutes for abdominal radiography. However, the sample of pediatric patients was very reduced, thus making generalizations difficult; also, both techniques are very expensive^19^ ^,^ ^21^. 

Administration of an indigo carmine solution (sky blue) to check the positioning of the gastric tube is only useful when it is possible to ensure correct positioning of an anterior tube. In the study investigating this method^25^, the first passage of GT was always verified by radiological imaging, and subsequent exchanges were performed every three weeks. At the time of each change before the tube was removed, the techniques for verifying the presence of gastric secretion and pH were used to confirm the positioning. The anterior tube measurement was maintained for insertion of the new tube.

## Discussion

The first description found in the literature on NEX and NEMU methods dating from 1978 was not included in this review, as it did not clearly present the method described. In this study, the authors describe using the NEX measure in clinical practice, however, they suspected that it was not a long enough measure, as they were not always able to aspire gastric contents. In order to validate their hypothesis, the authors followed some necropsies (they do not describe how many), and observed that with the NEX method, the distal end of the tube was at the limit of the gastroesophageal junction, and that it was necessary to add a few centimeters to the measurement for the distal end of the tube to reach the body of the stomach. Thus, the authors proposed the NEMU method and observed that the tube was correctly positioned in necropsies using this method^29^. 

After this one, other studies have showed the inferiority of the NEX measure compared to the NEMU^12^ ^,^ ^18^ ^,^ ^20^. Although the latter also represents a measure that has high variability, the present review indicates that it is the best evidenced method to date to be reproduced in clinical practice.

Equations that use height^18^ ^,^ ^20^ and weight^22^ ^,^ ^27^ to calculate the gastric tube insertion measure seem to reproduce reliable results; however, the absence of experimental studies with such methods impedes them being used as a single reference. Therefore, it is suggested that these equations are only used as a supporting measure in the decision on the tube length to be introduced, at least until studies with new evidence are available.

For the population of NBs below 1,500g, use of the minimum length table of the tube to be introduced can also be indicated as an auxiliary method to avoid positioning above the gastroesophageal junction^13^. It should be noted that this table should only be used for the oral route of insertion. 

Verifying GT positioning in NBs is a process that requires nurses’ attention due to the unavailability of precise techniques such as electromagnetic tracings or diaphragm electrical activity evaluation, as well as the impossibility of performing a radiological examination at each tube exchange due to the costs and risks involved^1^ ^,^ ^2^. Thus (and the findings of this review confirm), nurses must use several strategies simultaneously, with the objective of increasing the safety of the procedure. 

The most easily accessible indicator is gastric secretion return to the tube aspiration, which presented good results in the accuracy tests of one of the reviewed studies^24^. Recommendations from international agencies^30^ ^-^ ^32^ also indicate pH (<5.0) evaluation of aspirated secretion as a technique for verifying GT positioning. Other studies^15^ ^-^ ^16^ suggest that combining pH assessment with secretion coloration (whitish, translucent, greenish or brownish) makes the assessment even safer, since these are the indicators with the best results among the accuracy tests.

The use of gastric shields (histamine-2 receptor antagonists and proton pump inhibitors), as well as continuous infusion of milk formula and the use of sterile water to wash the catheter raise questions about the safety of the aforementioned combined evaluation, since they could increase gastric pH^2^. However, the reviewed studies comparing gastric pH in NBs and infants did not find significant differences between those who received and did not receive these medications, as well as those who were fed continuous infusion, gavage, or those who underwent fasting^2^ ^,^ ^16^ ^,^ ^24^ ^,^ ^26^.

In the absence of gastric secretion return, the risk of improper placement increases. In this situation, nurses may insist on obtaining a sample, performing movement maneuvers with the newborns and injecting air (not more than 2ml). Since it is possible that the tube is in direct contact with the mucosa, these maneuvers can favor its displacement and attainment of secretion. If it is still not possible to aspirate secretion through the catheter after such maneuvers, the possibility of changing the catheter or performing a radiological examination can be discussed to visualize the path and positioning of the distal end^31^. 

The use of abdominal ultrasonography to verify GT placement has been shown to be a useful and effective technique in adults with high sensitivity and specificity; attaining 98.3 and 100%, respectively, when compared with the results of conventional radiological examination^33^. Its use in verifying the location of the end of the GT has been recommended in adult patients instead of radiological examination since it is a simple and fast technique, in addition to the advantage of not exposing the patient to radiation^34^ ^-^ ^35^. A study carried out in two intensive care units with 14 neonatal and pediatric patients also demonstrated the efficacy of ultrasound to evaluate jejunal tube placement in these patients^36^. 

A pilot study published as a letter^37^, which was not part of this review sample, reports that the use of ultrasonography to verify GT positioning in NBs is not a reliable technique, as it was only possible in one of the 10 cases studied to visualize the distal end of the tube in the stomach. However, all had the gastric position confirmed by the pH test (<5.5)^37^. Considering the small sample size of the cited study and data that contradict promising results in adults, it is necessary to perform more research with ultrasound in NBs.

Despite care for tube maintenance not being the subject of this review, it should be pointed out that monitoring the external length can be used as a supporting measure in maintenance of tube placement and patient safety, especially when dealing with long-term tubes. In the description of an implementation protocol for tube maintenance in NBs^5^ and in an integrative review^38^, the authors recommend that the external length should be checked and recorded in the medical record and/or recorded on the tube in a visible manner, always confirming it before use. However, it is relevant to consider that keeping the external length stable does not eliminate the risk of internal displacement.

In this review, it was identified that the procedure of introducing air through the tube and auscultating the epigastric region is the second chosen method of American nurses to confirm gastric positioning^28^, which is also observed in the clinical practice of the authors considering their action and teaching fields. However, literature indicates that it is possible to listen to the air bubbles in the epigastric region, regardless of whether the end of the tube is located in the stomach, esophagus or respiratory tract. Therefore, the use of this technique is discouraged and should be banned^1^ ^,^ ^16^ ^,^ ^29^ ^-^ ^32^ ^,^ ^38^.

As a contribution to clinical practice, the findings of this integrative review support, recommending the use of the NEMU method (with possible confirmation by the use of formulas based on weight or height) in order to reduce risks and complications related to the procedure since it presents a smaller proportion of error, and the combined performance of positioning verification techniques prior to each GT use (gastric secretion aspiration with pH and color assessment). 

Another integrative literature review^38^ addressing this subject was found, however, it also included pediatric patients up to 18 years of age. We also found literature reviews^1^ ^-^ ^2^ that did not present a detailed description of the method and included studies. Thus, the difference in the present integrative literature review was to gather evidence on the methods for measuring and confirming GT placement in NBs. Among the 17 studies of this integrative review, only one well-delineated randomized controlled clinical trial, two randomized controlled trials and 14 descriptive studies were found. No systematic reviews or meta-analyses were found.

Given the specificities of the age group in question and gaps in the literature, it is considered relevant to emphasize that there is a need for experimental research on the methods already described for measuring the tube and verifying its positioning in order to offer support and safety to neonatal clinical practice, as well as for the technological development of devices with affordable cost. 

The results of the present study were limited by the lack of research that specifically focused on neonates, as well as by the predominant number of descriptive studies which made it impossible to synthesize findings with high levels of evidence to innovate clinical practice.

## Conclusion

Regarding methods for measuring gastric tube for insertion in newborns, implemented morphological distances present high variability, which compromises their reliability. The use of the NEX measurement greatly increases the risk of positioning the tube tip above the gastroesophageal junction, and should be replaced by the NEMU measurement. New measurement methods based on weight and height have been developed, but clinical trials are still needed to test their efficacy. 

Regarding the choice of technique for placement verification after insertion, no other method is available as safe as the radiological examination of the chest and abdomen. The use of electromagnetic tracing seems promising and deserves further investigation in newborn subjects. However, it is still expensive and inaccessible in the Brazilian context. 

Evidence indicates that the absence of secretion return to tube aspiration is a simple and sensitive method, and therefore it should be seen as a strong indicator of inadequate positioning. Moreover, pH evaluation and secretion staining for verification of gastric placement are the indicators that present the best results in accuracy tests when compared with radiological examinations. 

Concerning implications for clinical practice, there is still a lack of evidence to establish safe protocols, as some current procedures should have already been abandoned as pointed out in the literature, such as the use of NEX for measuring the tube and epigastric region auscultation to confirm its positioning.
